# A role of metallothionein-3 in radiation-induced autophagy in glioma cells

**DOI:** 10.1038/s41598-020-58237-7

**Published:** 2020-02-06

**Authors:** Young Hyun Cho, Seung-Hwan Lee, Sook-Jeong Lee, Ha Na Kim, Jae-Young Koh

**Affiliations:** 1 Neural Injury Research Center, Asan Institute for Life Sciences, University of Ulsan College of Medicine, Seoul, Republic of Korea; 2 Department of Neurosurgery, Asan Medical Center, University of Ulsan College of Medicine, Seoul, Republic of Korea; 3 Department of Biomedical Sciences, University of Ulsan College of Medicine, Seoul, Republic of Korea; 4 Department of Bioactive Material Science, Jeonbuk National University, Jeonju, Jeollabuk-do Republic of Korea; 5 Department of Neurology, Asan Medical Center, University of Ulsan College of Medicine, Seoul, Republic of Korea

**Keywords:** CNS cancer, Macroautophagy, Mechanisms of disease, Cancer in the nervous system, Gliogenesis

## Abstract

Although metallothionein-3 (*MT3*), a brain-enriched form of metallothioneins, has been linked to Alzheimer’s disease, little is known regarding the role of *MT3* in glioma. As *MT3* plays a role in autophagy in astrocytes, here, we investigated its role in irradiated glioma cells. Irradiation increased autophagy flux in GL261 glioma cells as evidenced by increased levels of LC3-II but decreased levels of p62 (SQSTM1). Indicating that autophagy plays a cytoprotective role in glioma cell survival following irradiation, measures inhibiting autophagy flux at various steps decreased their clonogenic survival of irradiated GL261 as well as SF295 and U251 glioma cells. Knockdown of *MT3* with siRNA in irradiated glioma cells induced arrested autophagy, and decreased cell survival. At the same time, the accumulation of labile zinc in lysosomes was markedly attenuated by *MT3* knockdown. Indicating that such zinc accumulation was important in autophagy flux, chelation of zinc with tetrakis-(2-pyridylmethyl)ethylenediamine (TPEN), induced arrested autophagy in and reduced survival of GL261 cells following irradiation. Suggesting a possible mechanism for arrested autophagy, *MT3* knockdown and zinc chelation were found to impair lysosomal acidification. Since autophagy flux plays a cytoprotective role in irradiated glioma cells, present results suggest that *MT3* and zinc may be regarded as possible therapeutic targets to sensitize glioma cells to ionizing radiation therapy.

## Introduction

Patients with glioblastoma, the most malignant form of glioma, usually succumb within a few years after the diagnosis despite aggressive chemo and radiotherapies following surgery^1^. Such disheartening therapeutic outcomes desperately call for more effective measures. However, untoward biological characteristics of glioma such as aggressive local invasion and resistance to anticancer therapy, present a daunting obstacle in achieving this goal.

Dysregulation of autophagy has been implicated in cancer cell proliferation, invasion, high histological grades, and poor prognosis in a wide spectrum of cancers. These findings highlight the possibility that autophagy-related molecules may prove useful as prognostic markers and/or therapeutic targets. In addition, many of anticancer drugs along with ionizing radiation (IR) are known to activate autophagy in cancer cells. The functional significance of autophagy activation associated with cancer therapeutics is still controversial. A growing body of evidence supports the possibility that autophagy plays a self-defensive cytoprotective role against toxicity induced by anti-cancer therapies^2–4^, although opposing data favoring the cytotoxic effect of autophagy are also available^2,3^. Despite these controversies, the current consensus is that the modulation of autophagy may have significant effects in anti-cancer therapeutics. Although a number of intrinsic or extrinsic pathways of autophagy have been delineated, the final destination of all these pathways is the lysosome, where degradation of cargo contents occurs. For this reason, diverse attempts have been made to maximize cancer cell death following the treatments through reducing the rate of autophagy flux with drugs acting on lysosomes^4,5^. For instance, currently, several clinical trials are investigating the effect of the combination treatment with lysosomal inhibitors such as chloroquine (CQ) or hydroxychloroquine (HCQ) and various anticancer drugs or IR in various cancer types (http://clinicaltrials.gov).

Recent evidence indicates that aberrancy in zinc homeostasis is linked to a variety of cancers including breast, esophageal, gastric, colon, nasopharyngeal, prostate, bladder, and skin cancers^6^. As regard to the mechanistic roles of zinc dyshomeostasis in cancer cell proliferation, well known effects of zinc on transcriptional regulation of genes involved in cell growth, proliferation, and apoptosis, and on the oxidative stress have been suggested as such. Since zinc is involved in so many cellular processes, there exist a number of zinc binding proteins in cells. Of many zinc-binding proteins, metallothioneins (*MTs*) are regarded as the major regulators of cellular zinc. Of four isoforms (*MT1*–4), the ubiquitously expressed *MT1* and *MT2* have been most extensively investigated and frequently found to be overexpressed in many cancers such as breast, ovarian, renal, prostate, lung and colorectal cancers as well as soft tissue sarcomas, and associated with poor patient prognosis^6,7^. On the contrary, relatively little is known regarding the role of *MT3*, a CNS-enriched Mt isoform, in cancers including glioma. Unlike *MT1* and *MT2*, the status of *MT3* expression has been found rather inconsistent among different cancer types, upregulated in some cancers (i.e. breast, prostate, urinary bladder, and lung cancers)^8–12^, while downregulated in others (i.e. gastric and esophageal cancers and leukemia)^13–15^. Moreover, data regarding the role of *MT3* are still scant and contradictory, some suggesting malignant phenotype-promoting^8,9,11,12^ or others antitumor effect^15^ of *MT3*. As for glioma, induced expression of *MT3* as well as *MT1* and *MT2* following arsenic trioxide treatment on U87-MG glioblastoma cells was demonstrated^16^, which may be postulated as a potential mechanism for glioma resistance. Importantly, Mehrian-Shai *et al*.^17^ recently reported that, in glioblastoma patients, high expression of including *MT3* was associated with poor patient survival whereas low *MT* levels corresponded to good prognosis, suggesting the prognostic implications of Mts in glioma.

Since *MT3* was first identified as a neuronal growth inhibitory factor (GIF) that was deficient in brain extracts of Alzheimer’s disease^18^, altered *MT3* expression has been also reported in various neurological disorders such as Parkinson’s disease, Amyotrophic lateral sclerosis (ALS), Down syndrome, and Creutzfeld-Jakob disease^19^. The role of MT3 in the CNS pathologies appears to be either neuroprotective or cytotoxic depending on the experimental models. The neuroprotective effect of MT3, which is presumably mediated by its metal chelating and antioxidative abilities, was observed in epileptic brain injury, cortical cryoinjury, and a mutant superoxide dismutase 1 mouse model of ALS^20–22^. On the other hand, the cytotoxic effect of MT3 has been also demonstrated; intracellular zinc released from MT3 may trigger neuronal and astrocytic cell death^23–25^. We have previously demonstrated that MT3 plays a key role in regulating the function of lysosomes in astrocytes, in a zinc- and actin-dependent manner, which effects are not shared by MT1 and MT2^23^. Of note, free zinc may contribute to autophagic flux in neurons and astrocytes, as evidenced by the result that oxidative stress induces accumulation of zinc ions in autophagosomes and lysosomes, and the inhibition of zinc accumulation by chelators or MT3 silencing blocks the increases in autophagy flux^23,26,27^. Although detailed information as to how zinc increases autophagy flux, one possible mechanism may be that lysosomal zinc accumulation is correlated with subsequent lysosomal acidification^28^. Lysosomal acidity has been found to be defective in neurodegenerative conditions such as Alzheimer’s disease, in which autophagy flux is arrested^29^.

Based on these findings, we hypothesized that glioma cells, as a cancerous counterpart of glial cells of developing brains, might utilize this novel cellular mechanism involving MT3 and zinc as a route for circumventing the toxicity of IR. Here, we explored this intriguing possibility.

## Results

### Increases in autophagy flux in irradiated GL261 glioma cells

**Figure 1:**
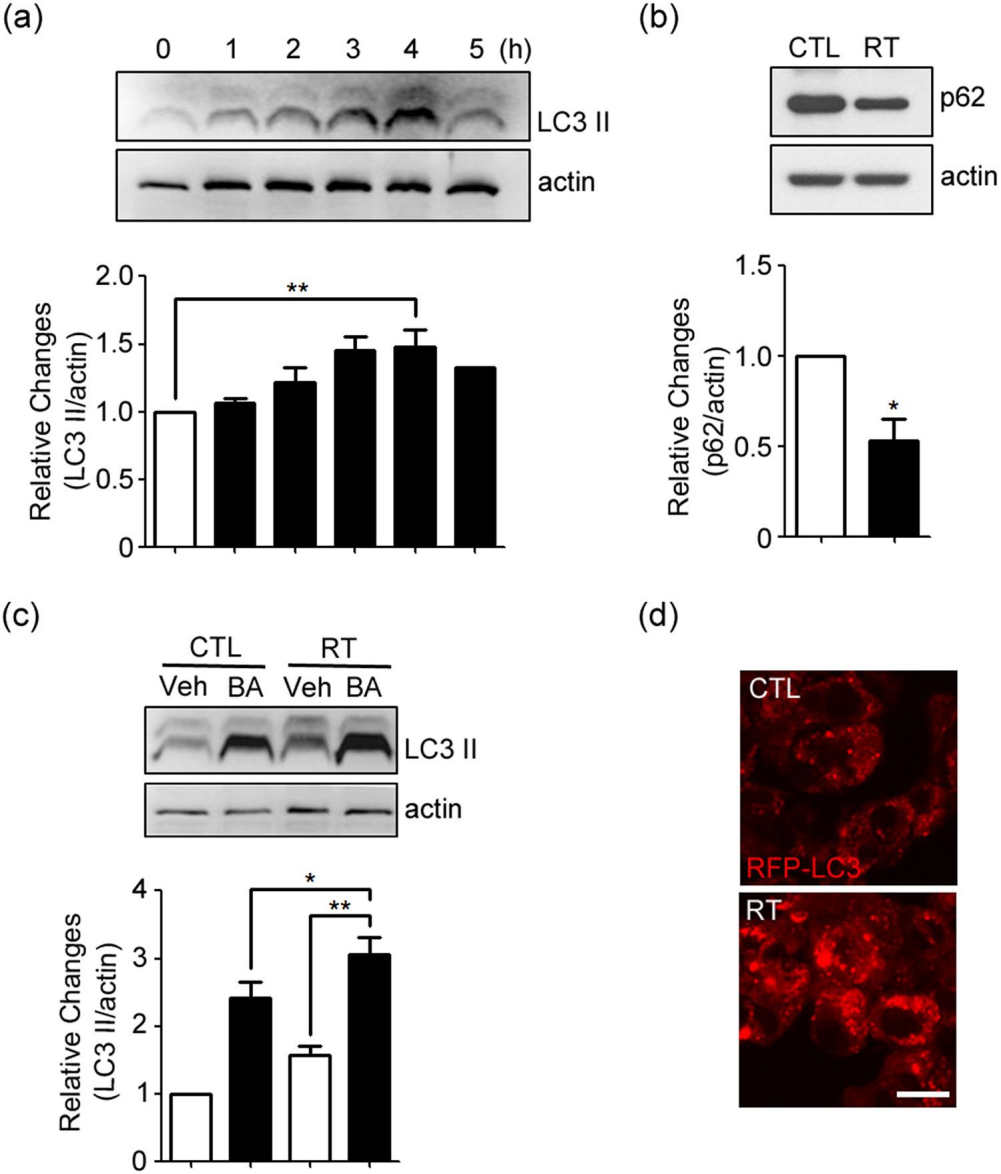
Induction of autophagy in irradiated GL261 cells. (**a**) Western blots for LC3 at 1 to 5 h after irradiation at 2 Gy. Western blot for β-actin is presented as a loading control. Bars denote the density ratio of LC3-II bands to the corresponding β-actin bands, normalized against the ratio in control (0 h) (mean ± SEM; *n* = 3 cultures; **P < 0.01 vs. 0 h). (**b**) Western blots for p62 (SQSTM1) 4 h after irradiation (RT) (mean ± SEM; *n* = 3 cultures; *P < 0.05 vs. control). (**c**) The increase in LC3-II levels after irradiation was accelerated when cells were treated with 50 nM BA immediately after irradiation (mean ± SEM; *n* = 3 cultures; *P < 0.05 vs. control with BA; **P < 0.01 vs. vehicle with RT). (**d**) RFP-LC3 fluorescence (red) in cells before (control) and 4 h after irradiation (RT). The number and intensity of RFP-LC3-positive vesicular puncta noticeably increased after irradiation. Confocal microscopic images were taken from a single Z-section. Scale bar: 10 μm.

To assess the autophagy flux in GL261 glioma cells after irradiation, we measured levels of LC3-II, a marker for autophagy activation, and p62 (SQSTM1), a marker for autophagic/lysosomal degradation. Immunoblots showed that levels of LC3-II gradually increased in GL261 glioma cells following irradiation at 2 Gy, reaching its peak level at 4 h and decreasing thereafter (Fig. 1a, see Supplementary Fig. 1(a) for the original blot). On the other hand, levels of p62 substantially decreased after irradiation (Fig. 1b, see Supplementary Fig. 1(b) for the original blot). Moreover, blockade of lysosomal degradation with bafilomycin A1 (BA) resulted in a further increase in LC3-II levels (Fig. 1c, see Supplementary Fig. 1(c) for the original blot), consistent with an increase in autophagy flux. Morphologically, confocal fluorescence microscopy showed that the number and intensity of RFP-LC3-positive vesicular puncta increased in the cytoplasm of RFP-LC3-transfected GL261 cells following irradiation (Fig. 1d). All these results indicated that irradiation increased autophagy flux in GL261 glioma cells.

### Inhibition of autophagy decreases clonogenic survival of irradiated GL261 cells

**Figure 2:**
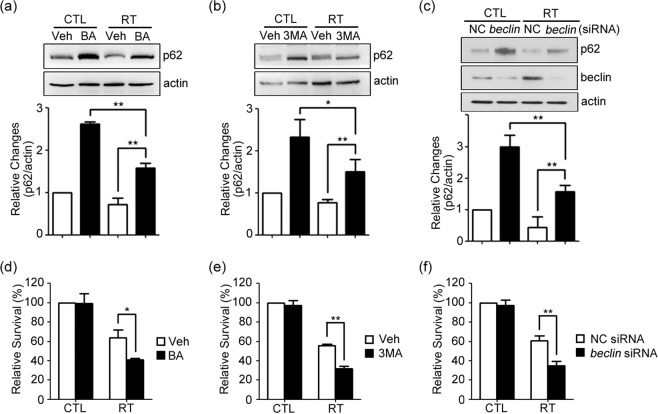
Inhibition of autophagy decreases clonogenic survival of irradiated GL261 cells. (**a**–**c**) Western blots for p62 4 h after irradiation at 2 Gy. Cells were treated with 50 nM BA (**a**) or 1 mM 3MA (**b**) immediately after irradiation, or transfected with *beclin* siRNA (*beclin*) or control siRNA (NC) for 24 h and then irradiated (**c**). Bars denote relative density of p62 bands normalized to corresponding β-actin bands in respective experiments (mean ± SEM; *n* = 3 cultures, each; **P < 0.01 vs. vehicle or NC with RT). (**d**–**f**) Cells were seeded at a density of 200 cells per well and allowed to attach overnight, and irradiated with treatment of BA (**d**), 3MA (**e**), or transfection with *beclin* siRNA (*beclin*) or control siRNA (NC) (**f**). The relative cell survival rates were calculated against the plating efficiency in control with vehicle (mean ± SEM; *n* = 3 or 4 cultures; *P < 0.05 or **P < 0.01 vs. vehicle or NC with RT).

To determine the functional significance of autophagy flux increased by irradiation, autophagy flux in irradiated cells was inhibited with pharmacologic inhibitors, BA or 3-methyladenine (3MA), or with siRNA against *Beclin1* (beclin). BA or 3MA was given immediately after irradiation, whereas siRNA was given 24 h prior to the irradiation. In each experiment, effective inhibition of autophagy flux was confirmed by an increase in p62 levels on Western blot analysis (Fig. 2a–c, see Supplementary Fig. 2(a–c) for the original blot). In colony forming assays, as compared with vehicle alone or negative-transfected controls (NC), cells treated with the drugs or *beclin* siRNA exhibited significantly lesser survival after irradiation (Fig. 2d–f). Since colony forming assay measures total cell number reflecting not only cell death but cell growth, we measured LDH efflux from dead cells as more direct measure for cell death. Also, we examined other human-derived glioma cell lines, SF295 and U251 cells. In all glioma cell lines, irradiation-induced cell death (LDH release) was increased by above measures inhibiting autophagy flux (Suppl. Fig. 6).

### siRNA knockdown of *MT3* blocks lysosomal degradation of autophagic vacuoles in irradiated GL261 cells and decreases cell survival

**Figure 3:**
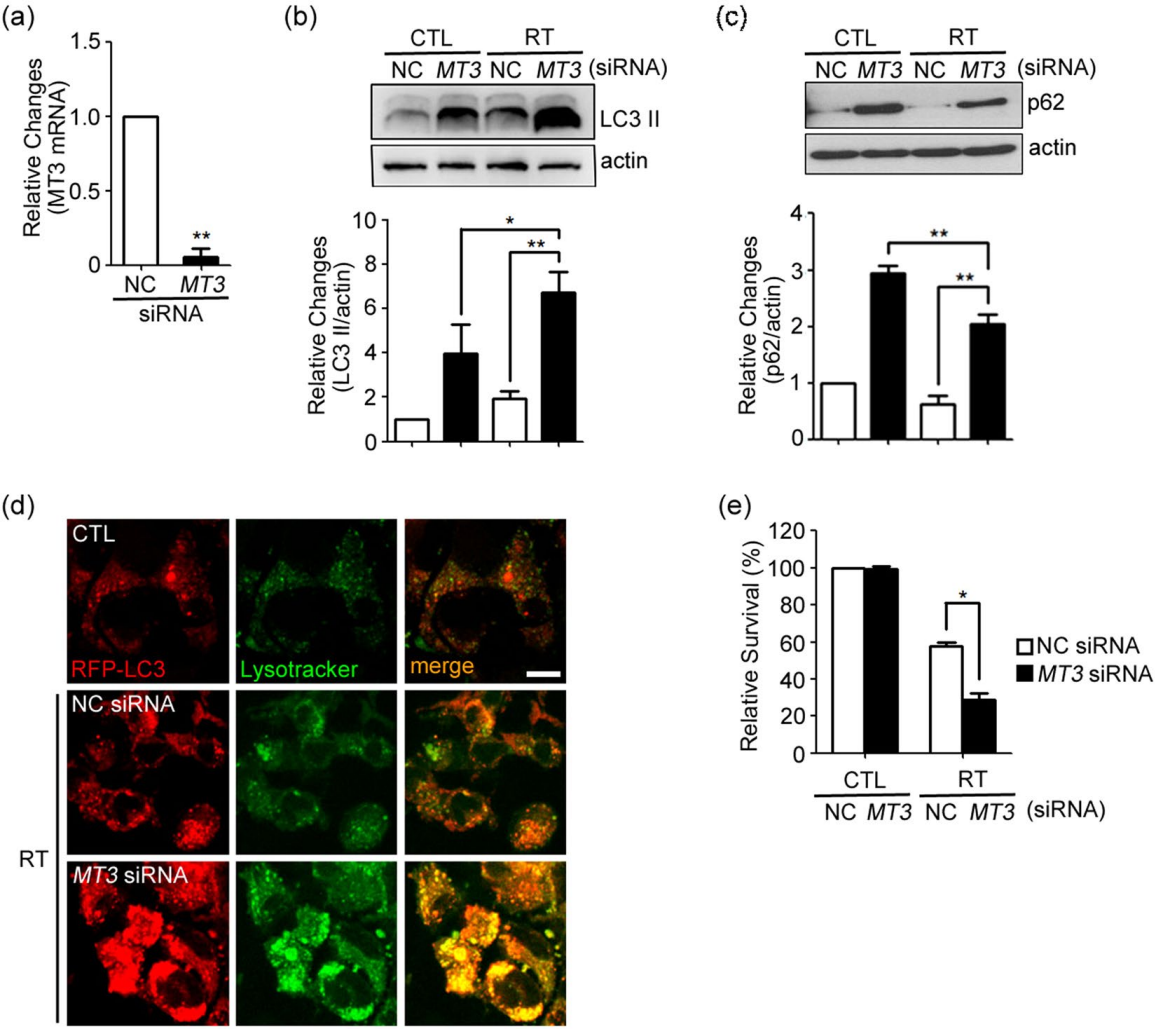
Knockdown of *MT3* blocks lysosomal degradation of AVs in irradiated GL261 cells and decreases cell survival. (**a**) Quantitative analysis of mRNA levels by RT-PCR for *MT3* after 24 h transfection with siRNA against *MT3*. Bars denote the ratio of MT3 mRNA values to the corresponding Gapdh mRNA values, normalized against the ratio in control siRNA (NC) (mean ± SEM; *n* = 3 cultures; **P < 0.01 vs. NC). (**b**,**c**) Western blots for LC3 (**b**) and p62 (**c**) 4 h after irradiation at 2 Gy in cells transfected with *MT3* siRNA or control siRNA (NC) (mean ± SEM; *n* = 4 cultures, each; **P < 0.01 vs. NC with RT). (**D**) Confocal fluorescence microscopic images of RFP-LC3-transfected cells stained with Lysotracker Green. Cells were transfected with *MT3* siRNA or control siRNA (NC), and irradiated. The fluorescences of RFP-LC3 and Lysotracker in *MT3* siRNA-transfected cells strikingly accumulated and overlapped 4 h after irradiation, indicating that lysosomal degradation of AVs was severely impaired. Images were taken from a single Z-section. Scale bar: 10 μm. (**e**) Clonogenic survival of irradiated cells transfected with *MT3* siRNA or control siRNA (NC). Bars denote the relative survival rates as described in Fig. 2 (mean ± SEM; *n* = 3 cultures; *P < 0.05 vs. NC with RT).

We have previously demonstrated that *MT3* plays a regulatory role in autophagy flux in primary cultured astrocytes^23^. To examine the role of *MT3* in GL261 glioma cell line, we downregulated *MT3* using siRNA (Fig. 3a). Compared with GL261 cells that were transfected with NC siRNA, GL261 cells that were transfected with *MT3* siRNA, showed increases in the levels of both LC3-II and p62 after irradiation (Fig. 3b,c, see Supplementary Fig. 3(b,c) for the original blot), suggesting that autophagy flux was inhibited distal to the autophagosome formation in the *MT3*-downregulated cells. To further confirm this, we observed RFP-LC3-transfected GL261 cells stained with Lysotracker Green under fluorescence microscope. In *MT3* siRNA-transfected cells, both RFP-LC3 fluorescence and Lysotracker fluorescence were increased in lysosomes (Fig. 3d), consistent with the possibility that lysosomal degradation of autophagic vacuole-associated RFP-LC3 was impaired. Subsequent survival analysis demonstrated that *MT3* knockdown decreased the survival of GL261 glioma cells after irradiation (Fig. 3e).

To confirm the finding, we prepared another siRNA to *MT3*. Both siRNAs reduced Mt3 mRNA levels (Suppl. Fig. 7a). The second siRNA also augmented radiation-induced cell death of GL261, SF295, and U251 glioma cells (Suppl. Fig. 7b).

### Accumulation of labile zinc in lysosomes following irradiation, which is attenuated by *MT3* knockdown

**Figure 4:**
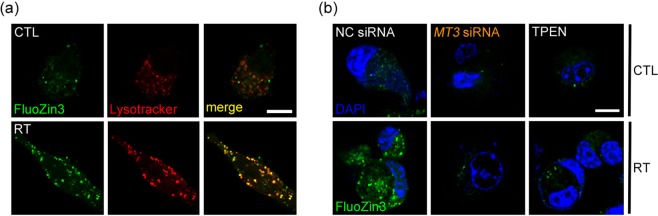
Accumulation of labile zinc in lysosomes following irradiation, which is attenuated by *MT3* knockdown. (**a**) Confocal fluorescence microscopic images of GL261 cells double-stained with FluoZin-3 and Lysotracker Red 2 h after irradiation at 2 Gy. The levels of labile zinc (green) increased in the cytosol after irradiation with its localization to lysosomes or autolysosomes (red) as its signal significantly overlapped (merge) with Lysotracker. Images were taken from a single Z-section. Scale bar: 10 μm. (**b**) Confocal fluorescence microscopic images of GL261 cells stained with FluoZin-3 (green) and 4′,6-diamidino-2-phenylindole dihydrochloride (DAPI; blue). Cells were transfected with *MT3* siRNA or control siRNA (NC), or treated with 1 μM TPEN, and irradiated. Scale bar: 10 μm.

As *MT3* releases zinc upon various stimuli such as oxidative stress, the fact that *MT3* knockdown reduced autophagy flux in irradiated GL261 cells raised a possibility that *MT3*-dependent zinc dynamics may be involved in this phenomenon. In line with our previous observation of zinc accumulation in lysosomes of neurons and astrocytes under oxidative stress^26,27^, irradiation of GL261 cells induced an elevation of labile zinc levels inside cells within 2 h as visualized with FluoZin-3 fluorescence microscopy; double staining of these cells with Lysotracker Red revealed that, most of zinc fluorescence was localized inside lysosomes (Fig. 4a). As expected from our previous study in cultured cortical astrocytes^23^, knockdown of *MT3* markedly attenuated the increase in labile zinc following irradiation (Fig. 4b). In addition, treatment with tetrakis-(2-pyridylmethyl)ethylenediamine (TPEN), a cell-permeant zinc chelator, almost completely blocked the increase in free zinc levels in lysosomes following irradiation (Fig. 4b).

### Chelation of intracellular zinc blocks lysosomal degradation of AVs in irradiated GL261 cells and decreases cell survival

**Figure 5:**
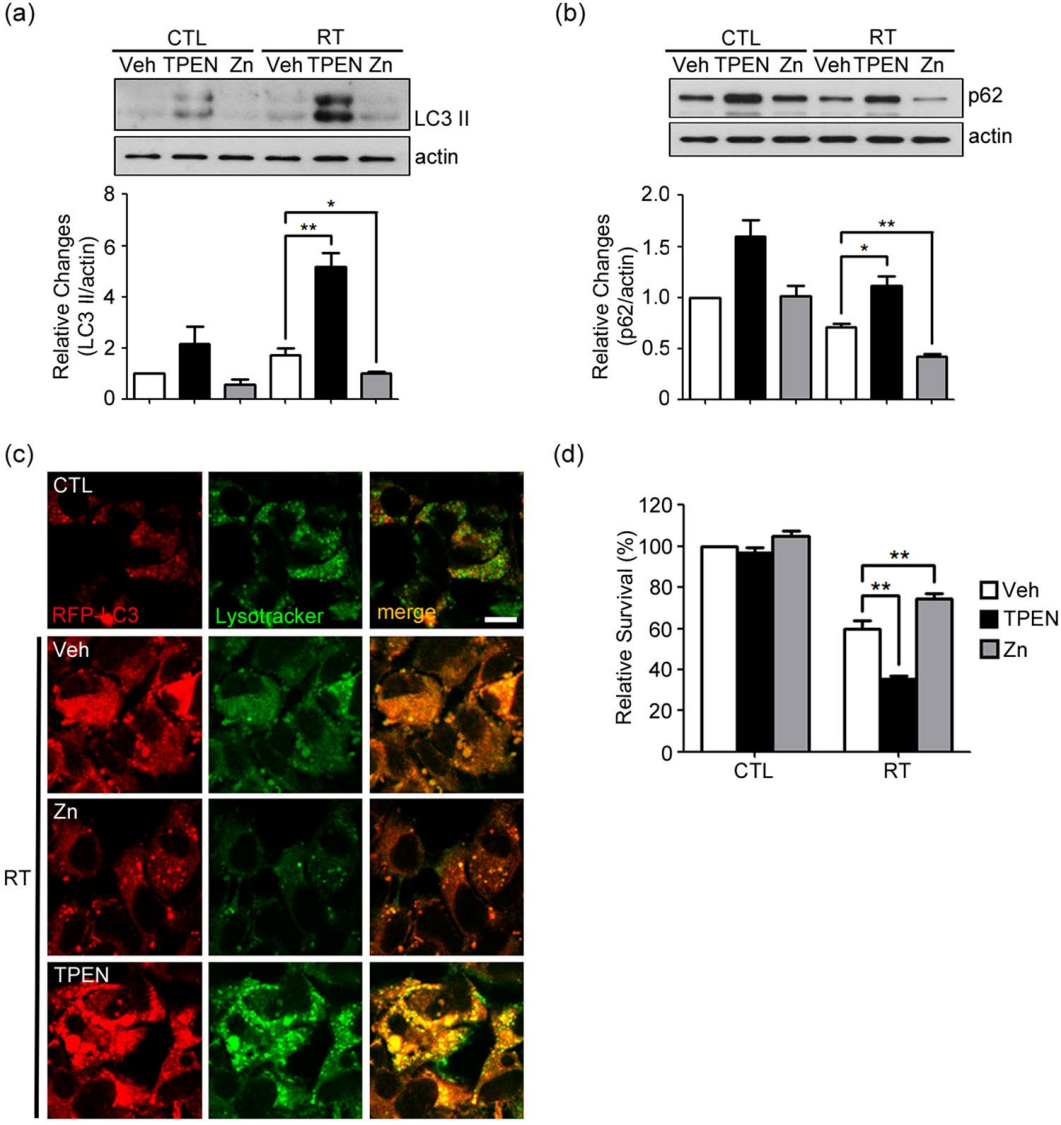
Chelation of intracellular zinc blocks lysosomal degradation of AVs in irradiated GL261 cells and decreases cell survival. (**a**,**b**) Western blots for LC3 (**a**) and p62 (**b**) 4 h after irradiation at 2 Gy in cells treated with 1 μM TPEN or 60 μM Zn (mean ± SEM; *n* = 3 cultures; *P < 0.05 or **P < 0.01 vs. vehicle with RT). (**c**) Confocal fluorescence microscopic images of RFP-LC3-transfected cells stained with Lysotracker Green. Cells were treated with TPEN or Zn, and irradiated. In TPEN-treated cells, a striking accumulation of dilated AVs mostly fused with lysosomes was noted 4 h after irradiation, which was concordant with the effect of *MT3* knockdown as seen in Fig. 3. In contrast, addition of Zn attenuated the accumulation of AVs after irradiation. Images were taken from a single Z-section. Scale bar: 10 μm. (**d**) Clonogenic survival of irradiated cells treated with 1 μM TPEN or 60 μM Zn. Bars denote the relative survival rates as described in Fig. 2 (mean ± SEM; *n* = 5 cultures; **P < 0.01 vs. vehicle with RT).

The two findings that labile zinc accumulates in lysosomes or autolysosomes following irradiation and that knockdown of *MT3*, a probable source of zinc, blocks degradation of AVs imply that zinc may have a direct effect on the degradative function of lysosomes, and consequently on cell survival. To test this hypothesis, we examined the effects of depleting or adding labile zinc on these events. In TPEN-treated (zinc-depleted) cells, changes similar to those seen in *MT3*-downregulated cells were observed. First, the levels of both LC3-II and p62 markedly increased after irradiation (Fig. 5a,b, see Supplementary Fig. 4(a,b) original blot). Second, striking accumulation of dilated RFP-LC3 (+) AVs in lysosomes was observed (Fig. 5c). These findings indicate that lysosomal degradation of AVs was reduced, and hence autophagy flux was arrested as in the case of *MT3*-knockdown cells. The clonogenic survival of TPEN-treated cells were also reduced after irradiation (Fig. 5d). In contrast to TPEN treatment, addition of zinc chloride (Zn) in the media, increased autophagy flux and lysosomal degradation as indicated by decreased levels of both LC3-II and p62 together with attenuated accumulation of AVs after irradiation (Fig. 5a–c). As expected, raising intracellular zinc with zinc treatment that increased autophagy flux, resulted in the increased cell survival following the irradiation (Fig. 5d).

### Impaired lysosomal acidification by *MT3* knockdown and zinc chelation in irradiated GL261 cells

**Figure 6:**
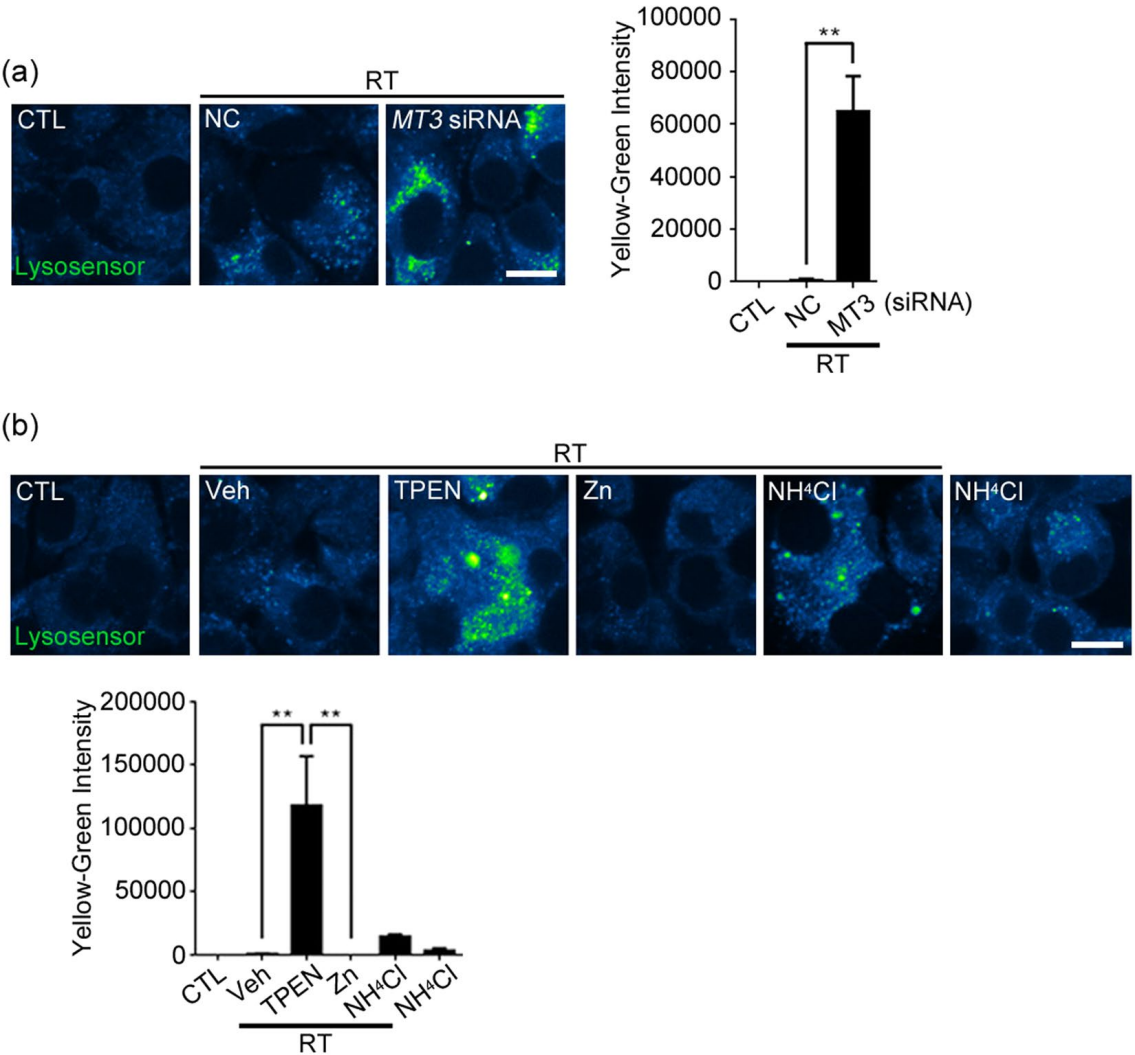
Impaired lysosomal acidification by *MT3* knockdown and zinc chelation in irradiated GL261 cells. (**a**,**b**) Confocal fluorescence microscopic images of irradiated cells stained with a lysosomal pH indicator dye Lysosensor Yellow/Blue dextran (Lysosensor). Cells were transfected with *MT3* siRNA or control siRNA (NC) (**a**) or treated with 1 μM TPEN, 60 μM Zn, or 60 μM NH_4_Cl (**b**), and irradiated at 2 Gy. The emission 510 and 450 nm were assigned the colors blue and yellow, respectively. The less acidic the vacuoles are, the lower the Blue/Yellow ratio is. Note the accumulation of yellow-green fluorescences in irradiated cells transfected with *MT3* siRNA or treated with TPEN or NH_4_Cl. Images were taken from a single Z-section. Scale bar: 10 μm.

In diverse cases of arrested autophagy, lysosomal acidification has been found to be abnormal. To examine this possibility, changes in lysosomal pH were assessed using a fluorescent lysosomal pH indicator dye Lysosensor Yellow/Blue dextran. Consistent with the result showing that *MT3* knockdown induced arrested autophagy in GL261 glioma cells, following irradiation *MT3* deficient glioma cells exhibited lysosomal pH shifting toward more alkaline direction as compared with that in Negative control transfected cells (Fig. 6a). Similar changes (“alkalinization”) in lysosomal pH following irradiation were observed in zinc-depleted (TPEN-treated) or NH_4_Cl-treated glioma cells (Fig. 6b). These results support the possibility that zinc and *MT3* play a critical role in the maintenance of acidity of lysosomal lumens in these cells.

## Discussion

While autophagy has been implicated as an important factor in glioma cell biology, its precise role in radio-sensitivity of glioma has been still controversial as to the end result being beneficial or harmful. The current study presents novel insights into this issue. First, present results demonstrated that *MT3*, possibly by releasing zinc that enters lysosomes, is a key regulator of autophagy flux that changes in response to irradiation. Second, thusly enhanced autophagy flux may be cytoprotective and increase the survival of glioma cells after irradiation. Therefore, downregulation of *MT3* or zinc chelation at the time of irradiation treatment may provide a beneficial effect to glioma patients by blocking the autophagy flux and increasing glioma cell death. Mechanistically, it appears that irradiation releases zinc from *MT3*, which in turn causes lysosomal acidification and increases autophagy flux and lysosomal degradation. These results are in line with the glioma clinical trials with the lysosomal acidification inhibitors, CQ and HCQ^30^, which have shown a modest efficacy^31–35^. Present results further support this idea, and suggest that the strategy aiming at the blockade of lysosomal acidification, especially targeting *MT3* and/or zinc, may prove useful to find a supportive measure to current glioma therapy.

In the present study, to examine the effect of autophagy on irradiation-induced death of GL261 cells, we modulated the autophagy flux by several different methods. First, we used standard autophagy inhibitors such as 3MA, BA, and siRNA against *beclin*. All these measures inhibited autophagy flux, and resulted in the increased death of GL261 glioma cells following irradiation. Two other human glioma cell lines, SF295 and U251 cells, showed the same effects. Next, we examined the effects of *MT3* knockdown with siRNA as well as altering intracellular zinc levels with TPEN or zinc. As *MT3* knockdown or zinc depletion produced similar results as the above autophagy inhibitors, we concluded that *MT3* and zinc play a key role in positively modulating the autophagy flux.

*MTs* are a family of small (6–7 kd), intracellular, cysteine-rich proteins with a number of thiol (-SH) groups enabling them to bind metals such as zinc and copper as well as toxic heavy metals. In oncogenesis, *MTs* are generally regarded to function as tumor suppressors in normal cells via chelating carcinogenic metal ions and maintaining redox homeostasis. On the other hand, *MTs* are upregulated in diverse cancer cells, and their levels are positively correlated with the degree of malignancy and the resistance to anticancer therapy^36,37^. For example, high levels of *MTs* confer resistance to platinum chemotherapeutics, and hence are regarded as a marker for poor prognosis^6,7^. The upregulation of *MTs* may be a response of cancer cells to metabolic stresses associated with aberrant cancer cell biology, and may function as a defense against anti-cancer therapeutics.

There are 4 isoforms of *MTs*. Of these, *MT3* is expressed mainly in the CNS. With approximately 70% sequence homology to other *MTs*, *MT3* contains a unique conserved sequence TCPCP motif at positions 5–9 in the N-terminus. A characteristic conformational organization of the β domain by virtue of the TCPCP motif of *MT3* in its zinc-bound form is suggested to provide a potential interface for protein-protein interactions, which may be responsible for the distinct biological functions of *MT3* such as neuronal growth inhibitory activity^38,39^. Other biological functions of *MT3* include roles in glycolytic metabolism, protein chaperone and scaffolding functions, metal transport/buffering, and redox signaling^25,40,41^. We have previously demonstrated that *MT3* plays a role in the maintenance of autophagy flux in cultured cortical astrocytes, likely by regulating lysosomal functions^23^. Similar to the findings obtained in primary astrocytes, in the present study, siRNA knockdown of *MT3* in GL261 glioma cells induced arrested autophagy following irradiation, likely due to impaired lysosomal degradation. Our results indicated that release of free zinc from *MT3* may be a key event herein, since zinc chelation produced similar effects. Both *MT3* knockdown and zinc chelation induced significant alkalinization of lysosomes, which should inhibit acidic hydrolase activities in lysosomes. Conversely, supplementation of zinc induced lysosomal reacidification. Although it is unclear how intracellular zinc release and its accumulation in lysosomes help acidify lysosomes, one plausible speculation is that a certain Zn^2+^/H^+^ antiporter may be functioning in lysosomes. Hence, zinc inside lysosomes may provide energy for proton to enter the lysosomal lumen. Alternatively, zinc may indirectly upregulate the expression of lysosomal proton pumps such as the vacuolar-type ATPase. These intriguing possibilities remain to be tested.

The cytoprotective effect of *MT3* in glioma cells has been reported by others. For instance, elevated *MT3* expression in U87-MG glioblastoma cells than in normal astrocytes and other glioma cell line was correlated with an inactive conformational change of p53, resulting in attenuated apoptosis^17^. Hence, *MT3* may contribute to glioma survival through diverse ways including increases in autophagy flux through lysosomal acidification. Further studies will be needed to elucidate the comprehensive mechanisms underlying the cytoprotective effect of *MT3* on glioma resistance.

In summary, we demonstrated the roles of *MT3* and zinc in radiation-induced autophagy and radioresistance in glioma cells. *MT3* appears to be a key regulator for autophagy flux via zinc-dependent lysosomal acidification, and hence contributes to resistance of glioma cells to irradiation treatment. As such, *MT3* may prove to be a suitable target in improving the efficacy of irradiation treatment in gliomas.

## Materials and Methods

### Cell lines and cell culture

The mouse glioma cell line GL261, recognized as recapitulating many of the features of human glioblastoma^42^, was obtained from the Tumor Bank Repository at the National Cancer Institute (Frederick, MD, USA). The human glioma cell line U251 and SF295 was obtained from the Tumor Bank Repository at the National Cancer Institute (Frederick, MD, USA). The GL261 and U251 cells were cultured in Dulbecco’s modified eagle’s medium (Life Technologies, Grand Island, NY, USA) supplemented with 10% fetal bovine serum (Life Technologies) and antibiotics (100 IU/mL penicillin and 100 μg/mL streptomycin; Lonza, Allendale, NJ, USA) at 37 °C with 5% CO_2_. The SF295 cells were cultured in RPMI 1640 (Life Technologies) supplemented with 10% fetal bovine serum (Life Technologies) and antibiotics (100 IU/mL penicillin and 100 μg/mL streptomycin; Lonza, Allendale, NJ, USA) at 37 °C with 5% CO_2_.

### Irradiation

Cells were irradiated using a 6-MV photon beam linear accelerator system (Varian Medical Systems, Palo Alto, CA, USA). For radiation dose titration, a single-dose of 2, 5 or 10 Gy was tested for autophagy induction and cell survival. Having identified that 2 Gy of radiation was sufficient for autophagy induction and optimal for cell survival analysis (data not presented), 2 Gy was used for experiments.

### Reagents and antibodies

3-methyladenine (3MA), bafilomycin A1 (BA), tetrakis [(2-pyridylmethyl)ethylenediamine] (TPEN), zinc chloride (Zn), ammonium chloride (NH_4_Cl), leupeptin, and pepstatin A were purchased from Sigma (St. Louis, MO, USA). Anti-microtubule-associated protein light chain 3 (LC3) and anti-p62 antibodies were obtained from Novus (Littleton, CO, USA) and MBL (Nagoya, Japan), respectively. Anti-β-actin and anti-beclin1 antibodies were from Cell Signaling (Beverly, MA, USA).

### Plate colony forming assay

Cells were seeded in six-well plates at a density of 100 (for controls) or 200 cells (for irradiated cells) per well and allowed to attach overnight before irradiation. Fourteen days after irradiation, cells were fixed with 4% paraformaldehyde and stained with Giemsa staining reagent (Sigma). The number of colonies with > 50 cells was counted under a dissecting microscope. The cell survival fraction or relative survival rate was expressed in terms of the plating efficiency in control groups: the number of colonies formed after treatment divided by both the number of cells seeded and the plating efficiency. Each assay was performed in triplicate.

### LDH efflux assay for cell death

Cells death was quantitatively assessed by measuring lactate dehydrogenase (LDH) activity released into the culture medium from damaged cells, as described previously^43^. LDH concentrations were normalized to the mean concentration of cells exposed to 400 μM H_2_O_2_ (100%) after subtracting the mean value of sham-washed control cells (0%).

### Western blot analysis

Cells were washed with ice-cold PBS and lysed in PRO-PREP protein extraction solution (iNtRON, Sungnam, Korea). Cell lysates were separated on 12–15% SDS-PAGE gel and transferred to polyvinylidene difluoride membranes. The membranes were blocked and probed with antibodies against LC3, p62, beclin1 or β-actin for 12 h at 4 °C. After washing, blots were incubated with appropriate HRP-conjugated secondary antibodies (Cell Signaling) for 1 h at room temperature, and were washed. The blots were developed with SuperSignal West Pico Chemiluminescent Substrate kit (Thermo Scientific, Waltham, MA, USA). The band intensity was analyzed using ImageJ (NIH, Bethesda, MD, USA). All experiments were triplicated.

### Real-Time reverse transcriptase PCR

Total RNA was extracted from GL261, SH295 and U251 cells with Qiazol Reagent (Qiagen, Hilden, Germany). ImProm-2 reverse transcriptase kit (Promega, Madison, WI, USA) was used to generate cDNA according to the manufacturer’s instructions. Samples were subjected to real time PCR amplification using forward and reverse primers for *MT3*, LightCycler 480 SYBR Green 1 Master and LightCycler 480 machine (Roche, Mannheim, Germany). The following thermocycle conditions were used: an initial cycle at 95 °C for 5 min; 50 cycles, each at 95 °C for 10 s, 60 °C for 20 s, and 72 °C for 20 s; and a final cycle at 95 °C for 15 min. Melting curve analysis was performed on all PCR products to ensure that specific PCR products were generated. Data were analyzed using the comparative cycle threshold method. The levels of mRNA were normalized to those of the housekeeping gene Gapdh (glyceraldehyde-3-dehydrogenase), and expressed as relative fold changes.

### Confocal microscopy

1 × 10^5^ GL261 cells were seeded onto a 0.1 mg/mL poly-L-lysine-coated coverslip in 4-well plates and incubated for 12 h. After irradiation, cells were stained with 100 nM Lysotracker Red DND-99 or Lysotracker Green DND-26 (Invitrogen, Carlsbad, CA, USA) for 1 h or 10 μM FluoZin-3 (Invitrogen) for 30 min before fixation. For detection of changes in lysosomal pH, cells were stained with 0.5 mg/mL Lysosensor Yellow/Blue dextran (Invitrogen), a fluorescent lysosomal pH indicator dye, for 1 h. Then, cells were washed with PBS twice and were fixed with 4% paraformaldehyde. For counterstain, the fixed cells were stained with 4′,6-diamidino-2-phenylindole (DAPI). Coverslips were mounted with Fluoromount G (Southern Biotech, Birmingham, AL, USA), and were examined by LSM 710 confocal microscope (Carl Zeiss, Dublin, CA, USA).

### RFP-LC3 plasmid transfection

The RFP-LC3 expression plasmid was a generous gift from Drs. Maria Colombo and Michel Rabinovitch (Universidad Nacional de Cuyo, Mendoza, Argentina). Plasmid transfection was performed using Effectene (Qiagen) according to the manufacturer’s instructions. After 24 h transfection, cellular expression of RFP-LC3 was confirmed by Western blot analysis.

### Silencing of *beclin* and *MT3* genes

Small interfering RNA (siRNA) targeting for *beclin* was purchased from Thermo Scientific. Negative control siRNA and siRNAs targeting for *MT3* were from Genolution Pharmaceuticals Inc. (Seoul, Korea). Transient siRNA transfection into glioma cells was carried out using TransIT-TKO reagent (Mirus, Madison, WI, USA) according to the manufacturer’s instructions. After 24 h transfection with siRNA, the specific silencing was confirmed by real-time PCR or Western blot analysis as described elsewhere. Total RNA was reverse transcribed and the prepared cDNA was then subjected to PCR analysis using the following primer sets: Control siRNA (5′-CCUCGUGCCGUUCCAUCAGGUAGUU-3′, 5′-CUACCUGAUGGAACGGCACG-AGGUU-3′), siRNA targeting for *MT3* (GL261 cell) (5′-UAAAUCCCAUGCACAACAUUU-3′, 5′-AUGUUGUGCAUGGGAUUUAUU-3′), *MT3* #1 (5′-UAAAUCCCAUGAACAGCAUU-3′, 5′-UGCUGUUCAUGGGAUUUAUU-3′), *MT3* #2 (5′-GUGUGGCUGGUGUCCCCUU-3′, 5′-GGGGACACCAGCCACACUU-3′). Human cell line using MT3 #1 and #2 primer sequence.

### Statistics

All data presented are representative from at least 3 independent experiments. The numeric data are presented as means ± SEMs and evaluated using the Student *t* tests. *P* < 0.05 was considered statistically significant.

## Supplementary information

supplementary figures and legends.
