# Supplementary material for: A role of metallothionein-3 in radiation-induced autophagy in glioma cells
Source: Sci Rep. 2020 Feb 6;10:2015. doi: 10.1038/s41598-020-58237-7 (PMC7005189; doi:10.1038/s41598-020-58237-7)
Supplement: Supplementary file 1 — supplementary figures and legends. [file 41598_2020_58237_MOESM1_ESM.docx]

**A role of metallothionein-3 in radiation-induced autophagy in glioma cells**

Young Hyun Cho^1,2^, Seung-Hwan Lee^3^, Sook-Jeong Lee^1,4^, Ha Na Kim^1^, and Jae-Young Koh^1,5,^*

^1^Neural Injury Research Center, Asan Institute for Life Sciences, University of Ulsan College of Medicine, Seoul, Republic of Korea

^2^Department of Neurosurgery, Asan Medical Center, University of Ulsan College of Medicine, Seoul, Republic of Korea

^3^Department of Biomedical Sciences, University of Ulsan College of Medicine, Seoul, Republic of Korea

^4^Department of Bioactive Material Science, Chonbuk National University, Jeonju, Jeollabuk-do, Republic of Korea

^5^Department of Neurology, Asan Medical Center, University of Ulsan College of Medicine, Seoul, Republic of Korea

***To whom correspondence should be addressed:** Jae-Young Koh

Department of Neurology, Asan Medical Center, University of Ulsan College of Medicine, 88, Olympic-ro 43-gil, Songpa-gu, Seoul 05505, Republic of Korea

Phone: 82-2-3010-4127; Fax: 82-2-483-5446; E-mail: [jkko@amc.seoul.kr](mailto:jkko@amc.seoul.kr)

**
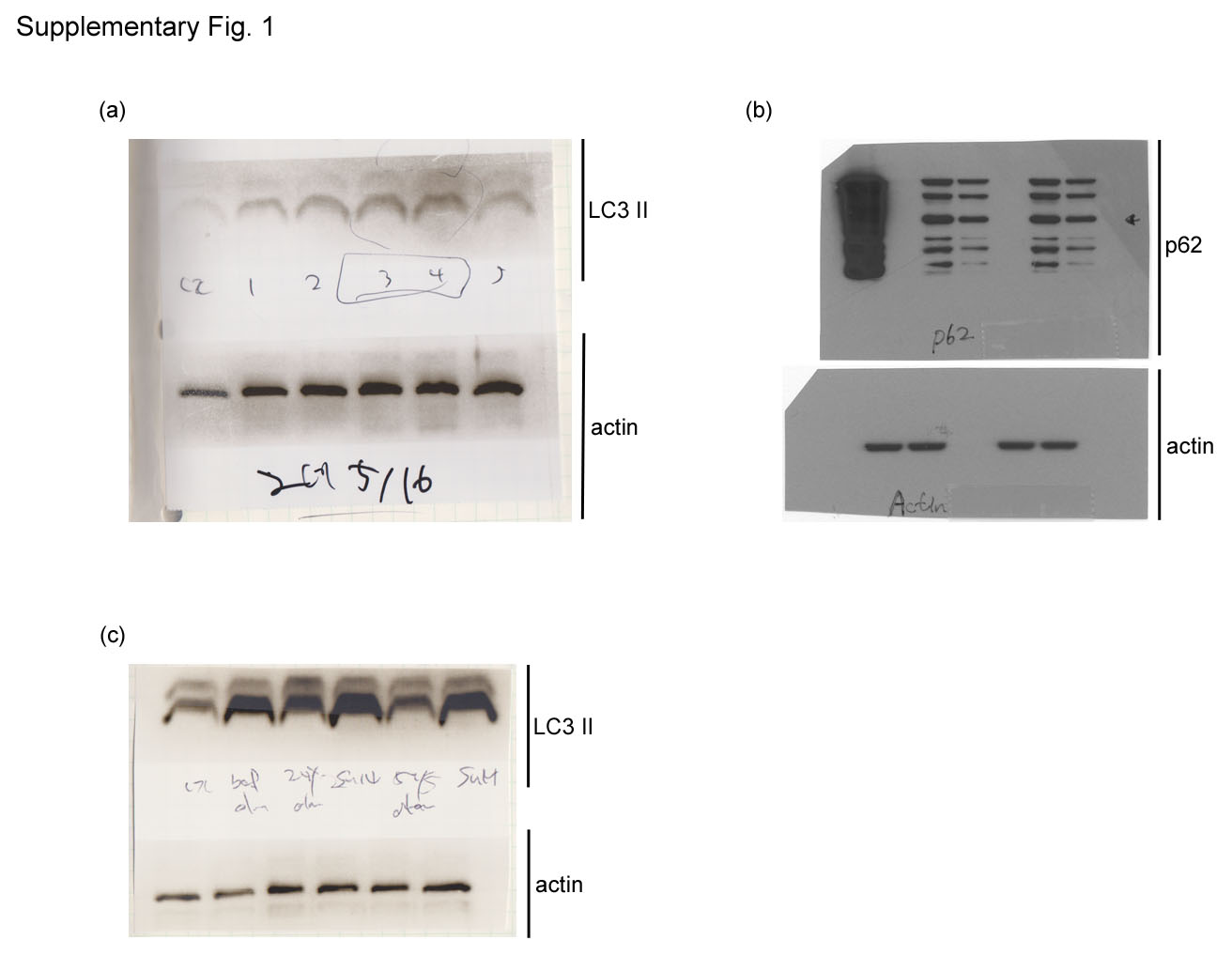
**

**Supplementary Fig. 1 Induction of autophagy in irradiated GL261 cells.**

(**a**) Original blot of western blots for LC3 at 1 to 5 h after irradiation at 2 Gy. (**b**) Original blot of western blots for p62 4 h after irradiation (RT). (**c**) Original blot of western blots for LC3 after irradiation (RT). The increase in LC3-II levels after irradiation was accelerated when cells were treated with 50 nM BA immediately after irradiation.

**
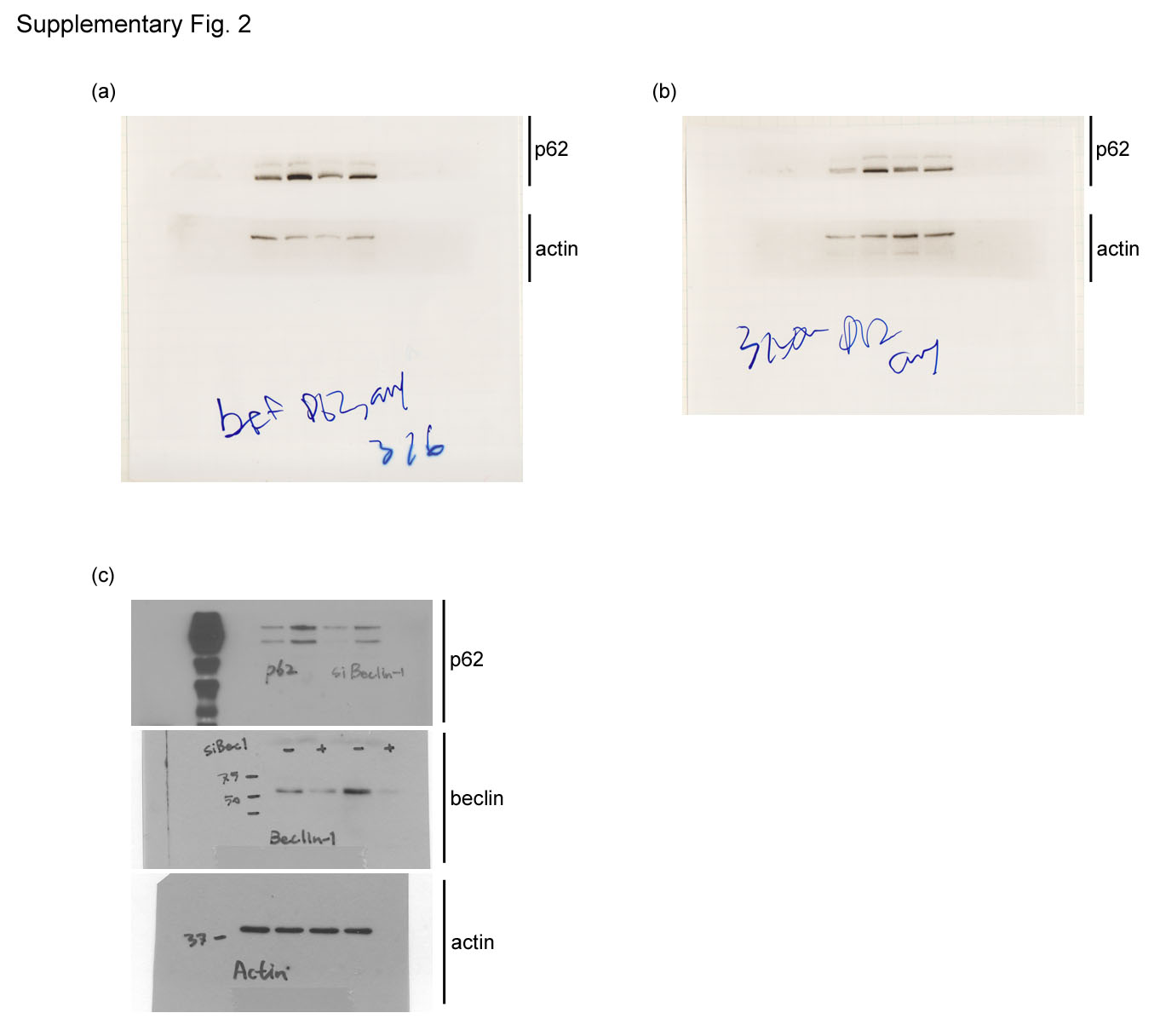
**

**Supplementary Fig. 2 Inhibition of autophagy decreases clonogenic survival of irradiated GL261 cells.**

(**a-c**) Original blot of western blots for p62 4 h after irradiation at 2 Gy. Cells were treated with 50 nM BA (**a**) or 1 mM 3MA (**b**) immediately after irradiation, or transfected with *beclin* siRNA (*beclin*) or control siRNA (NC) for 24 h and then irradiated (**c**).

**
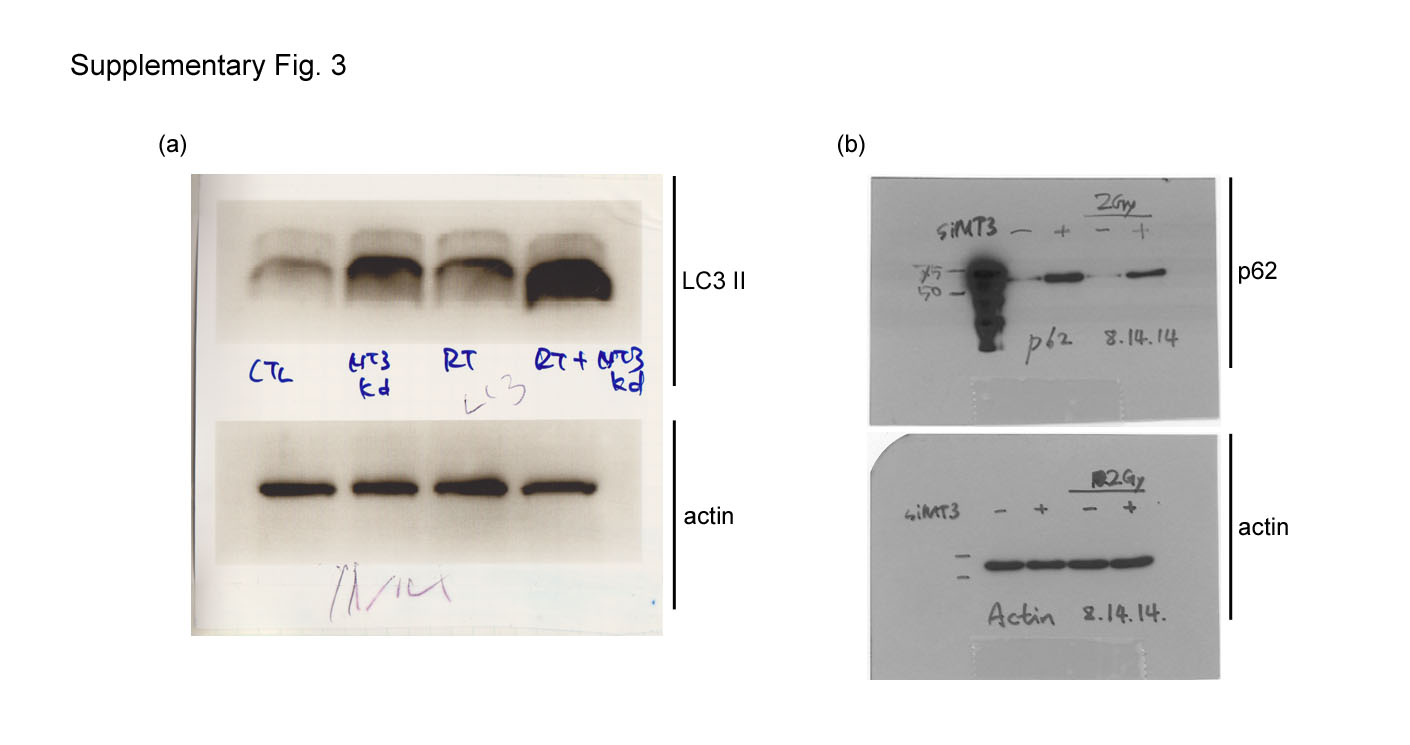
**

**Supplementary Fig. 3 Knockdown of *MT3* blocks lysosomal degradation of AVs in irradiated GL261 cells and decreases cell survival.**

(**a and b**) Original blot of western blots for LC3 (**a**) and p62 (**b**) 4 h after irradiation at 2 Gy in cells transfected with *MT3* siRNA or control siRNA (NC)

**
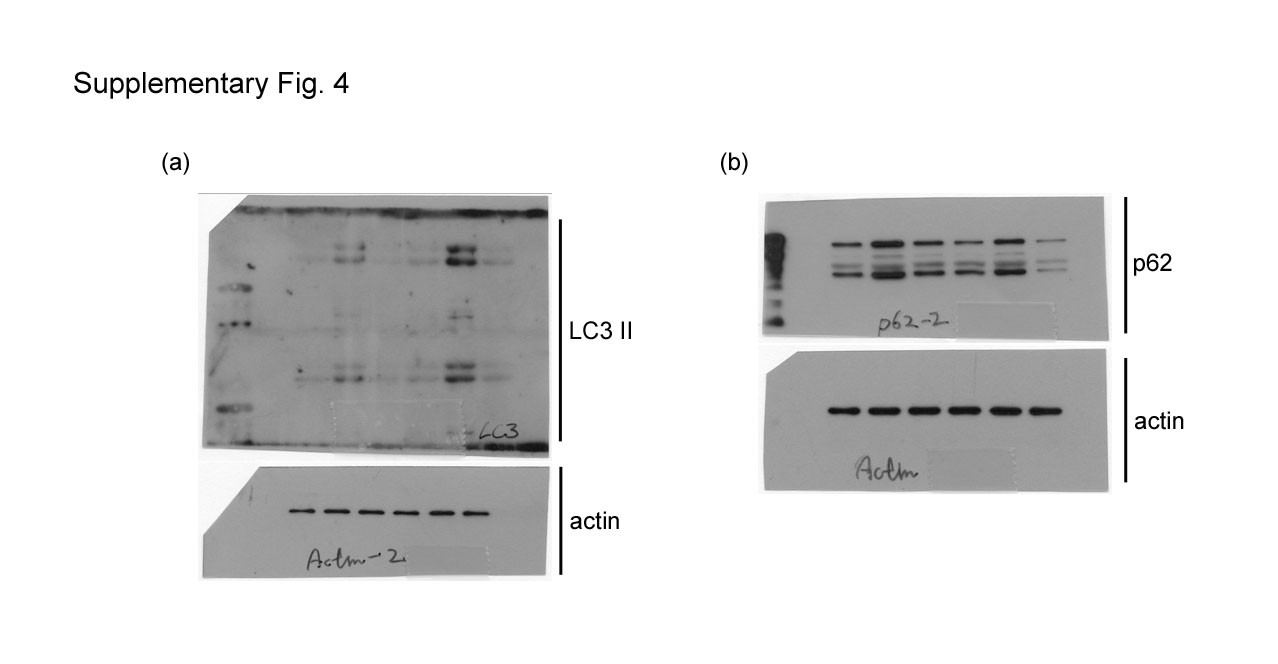
**

**Supplementary Fig. 4 Chelation of intracellular zinc blocks lysosomal degradation of AVs in irradiated GL261 cells and decreases cell survival.**

(**a and b**) Original blot of western blots for LC3 (**a**) and p62 (**b**) 4 h after irradiation at 2 Gy in cells treated with 1 μM TPEN or 60 μM Zn.

**
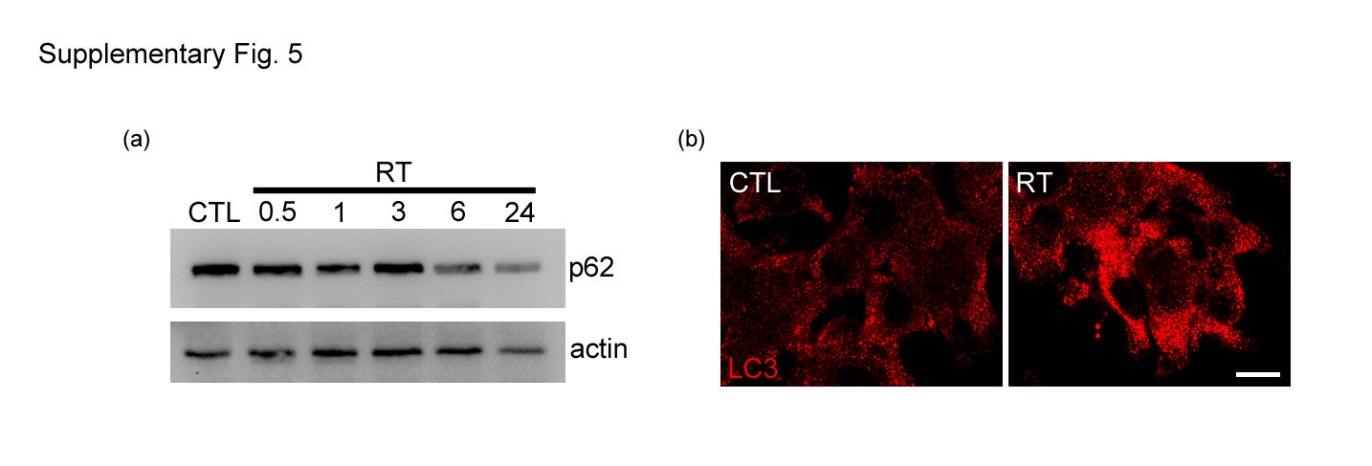
**

**Supplementary Fig. 5 Induction of autophagy in irradiated GL261 cells.**

(**a**) Original blot of western blots for p62 at 0.5 to 24 h after irradiation at 2 Gy.

(**b**) Confocal microscopic images of cells stained with LC3 fluorescence (red) before (CTL) and 4 h after irradiation (RT). The intensity of LC3 increased after irradiation. Scale bar: 10 μm.

**
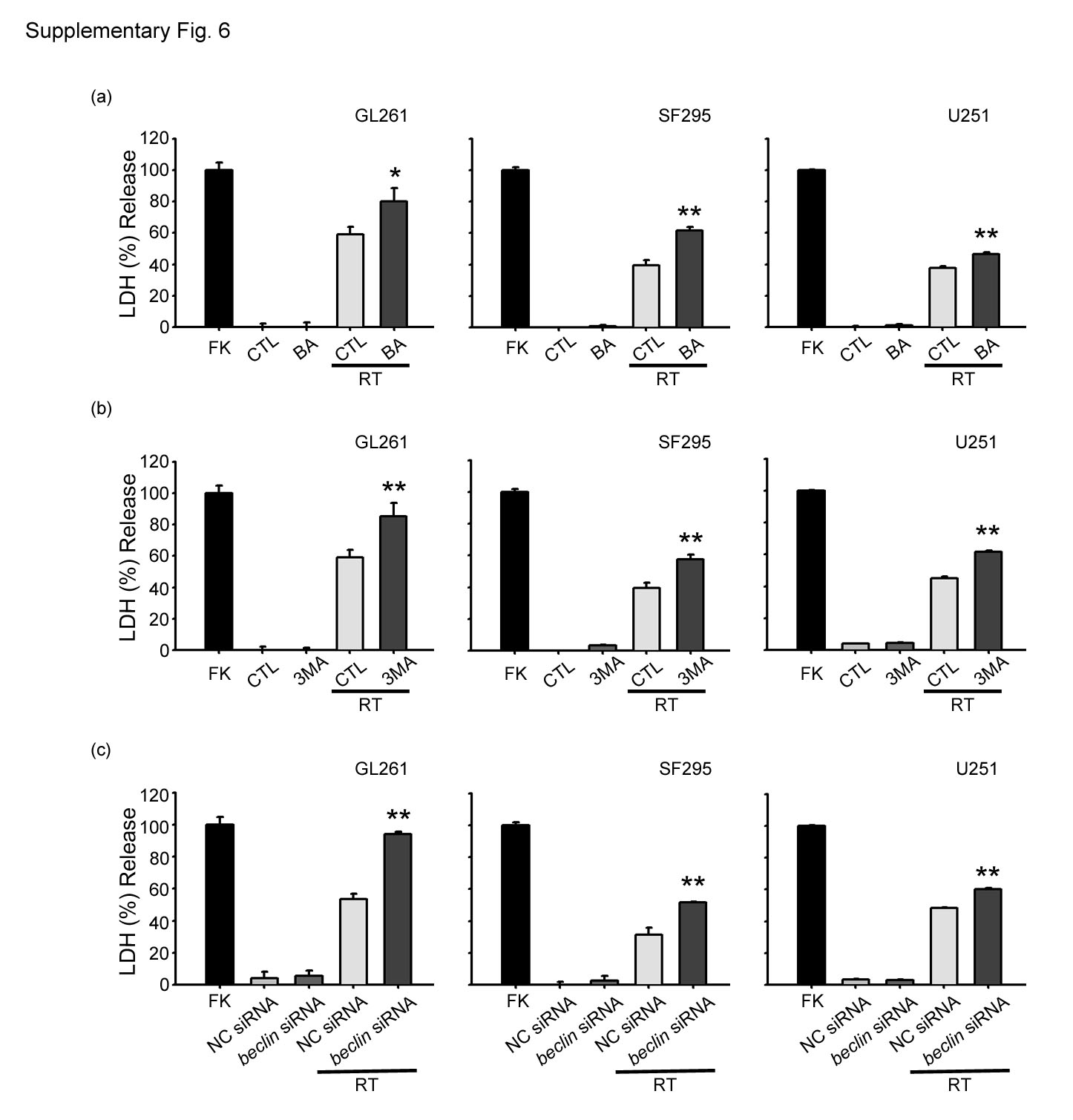
**

**Supplementary Fig. 6 Inhibition of autophagy decreases clonogenic survival of irradiated glioma cells.**

(**a-c**) Bar denote LDH release (mean ± SEM; n = 3) by glioma cells exposed to BA (a), 3MA (b), or transfection with *beclin* siRNA (*beclin*) or control siRNA (NC) (c) after irradiation at 2 Gy (mean ± SEM; *n* = 3 or 4 cultures; **P* < 0.05 or ***P* < 0.01 vs. vehicle or NC with RT).

**
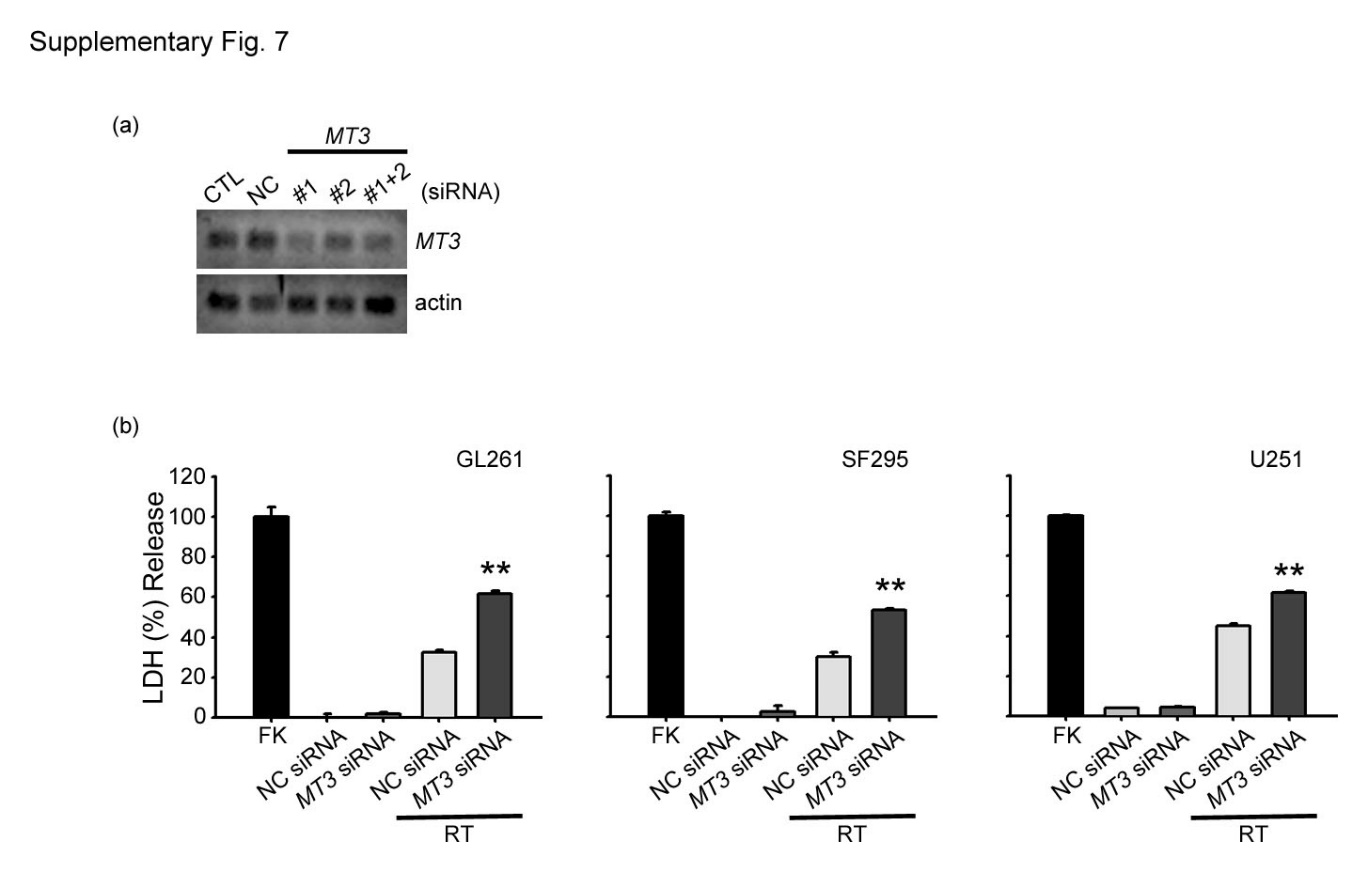
**

**Supplementary Fig. 7 Knockdown of *MT3* decreases cell survival in glioma cells.**

(**a**) mRNA levels by RT-PCR for *MT3* after 24 h transfection with siRNA against *MT3*. mRNA levels were determined after silencing two MT3 sequences.

(**b**) Bar denote LDH release (mean ± SEM; n = 3) by glioma cells exposed to transfection with *MT3* siRNA (*MT3*) or control siRNA (NC) after irradiation at 2 Gy (mean ± SEM; *n* = 3 or 4 cultures; **P* < 0.05 or ***P* < 0.01 vs. vehicle or NC with RT).
